# Virtual Overdose Response for People Who Use Opioids Alone: Protocol for a Feasibility and Clinical Trial Study

**DOI:** 10.2196/20183

**Published:** 2021-05-12

**Authors:** Sean Kristina Bristowe, Sumantra Monty Ghosh, Michael Trew, Canadian Mental Health Association - Calgary Region, Katherine Rittenbach

**Affiliations:** 1 Department of Psychiatry University of Calgary Calgary, AB Canada; 2 Department of General Internal Medicine University of Alberta Edmonton, AB Canada; 3 Canadian Mental Health Association-Calgary Region Calgary, AB Canada; 4 Department of Psychiatry University of Alberta Edmonton, AB Canada

**Keywords:** harm reduction, opioid crisis, overdose response, people with lived experience, peers

## Abstract

**Background:**

A recent quarterly report released by Alberta Health reports that on average 2.5 Albertans die every day from accidental overdose deaths, and that between April 1, 2020, and June 30, 2020, the province lost a total of 301 people. In Canada, between January 2016 and March 2020, a total of 16,364 people died due to opioid-related overdose. The World Health Organization reports that 70% of the 0.5 million deaths worldwide caused by drugs are related to opioid overdose. Although supervised consumption sites or safe injection sites have been shown to be effective in reducing the harms associated with the use of illicit substances and increasing uptake of addiction treatment and other health services, there is still significant stigma associated with them, and it is unlikely that all of the people who would benefit from supervised consumption service will ever access a site.

**Objective:**

To help prevent deaths in populations that cannot or will not access physical safer consumption services in Alberta, we propose to provide virtual (telephone-based) overdose response services, staffed by people with lived experience.The primary outcome for this study is uptake of the service as measured by the number of calls to the service. Secondary outcomes will include patterns of use of the phone line (days of the week and time of calls) and outcomes from the calls (number of emergency medical services dispatches for overdoses from the service and the results of those dispatches).

**Methods:**

This phase 1 clinical study is set to officially launch in early May 2020. The service will be available to up to 15 participants who self-disclose as using opioids unobserved and have given informed consent for both data collection and interviews. This group will have access to a toll-free telephone number and be invited to call when they plan to use opioids alone.

**Results:**

The analysis will include mixed methods. To improve the design of the service and ensure safety of all involved, quantitative data will be collected on phone calls and participant health care usage, while qualitative data will be collected from both participants and virtual overdose response operators

**Conclusions:**

This clinical trial aims to test the feasibility of a service that provides virtual overdose response in order to help prevent deaths in populations that cannot or will not access physical supervised consumption services in Alberta.

**Trial Registration:**

ClinicalTrials.gov NCT04391192; https://www.clinicaltrials.gov/ct2/show/NCT04391192

**International Registered Report Identifier (IRRID):**

DERR1-10.2196/20183

## Introduction

### Background

Between January 2016 and March 2020, a total of 16,364 individuals across Canada lost their lives to accidental opioid-related poisonings [1]. In 2016, Alberta recorded the third highest number of opioid-related deaths in Canada [2]. A recent report from Alberta Health states that the province totals since the beginning of the opioid crisis have reached upwards of 3139 [3]. The World Health Organization (WHO) reports that 70% of the 0.5 million deaths worldwide caused by drugs are related to opioid overdose [4]. Opioid misuse is one of the most pressing public health problems of our time. Why this has happened is unclear but appears to have come from a number of factors, with prescribing practices being of primary concern.

Alberta had the highest number of defined daily doses of opioids prescribed in 2016, with 7955 per 1000 population. This was over twice as high as that in Quebec (the province with the lowest number per 1000 population) [5]. This represents a small decrease in prescriptions from 2015; however, the high level of prescriptions might have led to an increase in both dependence and addiction in the population with a resulting increase in the related harms. The rate of emergency department visits in Alberta related to opioid use and substance misuse has increased by 41% from 2016 to 2019 [6].

It has been postulated that as opioid prescriptions have become more regulated and more restricted, there has been an increase in demand for black market opioids, and that demand has shifted towards stronger formulations [7,8]. The increase in fentanyl and analogues has a direct impact on overdose rates, as they have come to contaminate a majority of illicit substances. Recent testing demonstrated that 88% of all opioids sold on the street contained fentanyl and only 19% contained the expected substance in any detectable amount, creating a toxic drug supply [9].

In Alberta, to face this mounting overdose crisis, concerted efforts have been made. The Alberta government has increased funding for prevention, treatment, and harm reduction, while also creating new educational tools and convening the Minister’s Opioid Emergency Response Committee [10]. There is also work ongoing to educate physicians on safer prescribing practices. The Alberta government, along with Alberta Health Services and the community harm reduction organization, Alberta Community Council on HIV (ACCH), have all actively supported and run a provincewide community-based naloxone program that now dispenses between 8000 and 9000 kits to individuals at risk of overdose each month (internal report, May 2020). These same partners have worked together to successfully apply for federal approval to open multiple supervised consumption service sites in the province. This has included conducting needs assessments in multiple communities in Alberta and applying for and receiving federal exemptions from the federal government.

Supervised consumption service (SCS) locations (sometimes referred to as safe injection sites or safe consumption sites) have been an effective harm reduction technique available since the late 1980s [11]. They have been shown to reduce some of the harms associated with illicit substance use, lowering disease transmission rates and mortality from overdoses [12]. In 7 studies that evaluated overdose harms in people who use supervised consumption services, no death by overdose was reported, and in Vancouver specifically, a 35% reduction in lethal overdoses in the vicinity was reported (this report was prior to 2014, and the current overdose crisis was not a factor during the evaluation) [12]. Despite these interventions, presentations to the emergency departments for overdoses due to opioid poisonings have not declined, demonstrating that more tools are needed.

Unfortunately, even though SCSs have been shown to be very effective in reducing the harms associated with the use of illicit substances and increasing uptake of addiction treatment and other health services [12], there is still significant stigma associated with them. This is demonstrated by the call for a review of the evidence by the Ontario government that was based on vocal objections by the public and claims that SCSs increase drug use [13]. This stigma can make it difficult to establish new sites in neighbourhoods that are experiencing increased harms from illicit substance use. Moreover, it may also make people who use illicit substances reluctant to visit the sites for fear of being seen and identified as someone who uses illicit substances [11], which in turn can potentially impact their personal and professional lives. Stigma impacts visible minorities and women disproportionately and also deters people who are employed and housed from accessing SCSs [11,14].

Further, due to the fact that people are bringing illicit substances into the SCS, an SCS must have a federal exemption in order to legally operate. This approval process can take a significant amount of time and is a further barrier to the establishment of more locations. Due to these factors, it is unlikely that all of the people who would benefit from SCS will ever access a site. Our project addresses this health care gap.

Another barrier to accessing SCSs is the impact of geographic location. Physical SCSs are only statistically effective in reducing mortality within 500 meters of their location [15]. This is of particular significance as the most recent (fourth quarter of 2019) provincial data show that the majority of the opioid-related deaths in the province occur outside of an SCS service areas with suburban and rural communities in Alberta accounting for 81% of overdose-related deaths [3]. SCSs cannot be established in every neighbourhood, and, while multiple analyses demonstrate that SCS are cost effective [16], at an average operation cost of $2.9 million per year, it is cost prohibitive to create SCSs for many of our communities [17]. In Alberta, the gap in care is exacerbated by geographical realities, as there are many people in rural and remote communities for whom SCSs are not supportable. Other barriers to access SCS treatment include the following: access to inhalational supervised consumption services for those who smoke or inhale substances, limited hours of access to some SCSs [18], and management of clients who need to use a substance expeditiously due to substance withdrawal symptoms [19].

The current COVID-19 pandemic guidance suggesting all capable individuals self-isolate and practice social distancing has exacerbated the opioid crises [18]. With more individuals who use substances isolating alone and with the reduction in client capacity for existing SCSs, further innovative interventions are required to support clients.

### Evidence for a Technology-Based Approach

Although there is limited information on technology-based harm reduction services, recent literature has demonstrated that clients who are dependent on opioids may be more likely to be retained in opioid agonist treatment [20] when their treatment is offered primarily through telehealth services. According to regulations around opioid agonist treatment, these services are generally video calls through secure technology in a clinic. This demonstrates that clients who use illicit substances will use technology for treatment and suggests that they may also use it for harm reduction. Further, a review from 2015 [21] reported that all illicit drug or alcohol helplines in the published literature have started with moderate call rates but have experienced increases in the call rates, high satisfaction with the call lines, and no negative effects.

Additionally, there are new technologies and services within Canada that have recently launched, including the Lifeguard app and the Brave Be Safe app in the province of British Columbia and a grassroots volunteer run overdose prevention line in Hamilton, Ontario [22-24]. The Lifeguard App requires that people who use drugs push a button once they have used their substances, after which the app will sound an alarm after about a minute has elapsed. Clients using this app are required to push the button once the alarm has sounded; otherwise, emergency services will be contacted. Both the Brave Be Safe app and overdose prevention line require the client to stay on the phone with a volunteer.

### Study Plan

To help prevent deaths in populations that cannot or will not access physical SCSs in Alberta, which may disproportionately include women and those who are not able to self-inject [11], we propose providing virtual (telephone-based) overdose response services staffed by people with lived experience (PWLE). Due to COVID-19 and the recent changes in protocols for research, study staff will engage potential participants with the necessary physical distancing precautions and with the highest personal protective equipment standards.

### Study Objectives

The primary objective of the study is to establish the feasibility of a virtual overdose response service with PWLE operators.

The secondary objectives of the study will include understanding how the service is used (demographics, timing, number of emergency medical services [EMS] responses); determining the outcomes of calls to the service; and using feedback from clients, peer operators, 911 dispatch, and EMS services to determine improvements to service provision.

## Methods

### General Design

We designed a small open-label clinical study to demonstrate proof of concept that will follow Consolidated Standards of Reporting Trials (CONSORT) guidelines. We aim to recruit approximately 15 people who are currently using illicit substances, specifically opioids, and those who sometimes use these substances alone. The sample size of 15 balances pragmatic issues (difficulty in recruiting people who are actively using illicit substances) and the need to have a good sample of the population. These participants will be interviewed by the research coordinator (SB) prior to intervention initiation to determine baseline use, history of overdose, and current harm reduction activities. They will then be asked to call the intervention number if they are going to be using alone and the PWLE operator will follow the call (the call flow is documented in Multimedia Appendix 1). Additionally, PWLE will be asked to participate in weekly interviews where the research coordinator will ask about their experience on the phones and to disclose any perceived issues or suggestions for improving the service.

Each time a participant calls the number, the operator will gather (as part of the intervention) the address where the participant is located, their name or pseudonym, and a phone number that can be used as a call back number in case the call is disconnected. The phone line operator will then ask what they planned on using, the method of use, if the participant is using sterile supplies (and provide information on where they can get new supplies in their community), and if they have a naloxone kit (overdose reversal kit) available. They will then inform the participant that they will be checking in on them every 5-10 minutes and if they do not respond, they will call emergency medical services for them.

If the participant responds to each verbal prompt (calling their name) over a minimum of 30 minutes, the operator will let them know that they are disconnecting the call. The operator will offer to connect the participant to other health services, such as the location of new supplies, social services, addiction treatment, and opioid agonist therapy. If the participant fails to respond to a prompt (or the call is disconnected and is not able to be reconnected), the operator will contact 911 and the process for emergency services dispatch will be initiated.

If a participant calls the phone line and a virtual overdose response (VOR) operator is not available to answer their call, the call will go to voicemail where the participant will be prompted to leave their phone number and ID so they can be contacted as soon as possible by the next available operator. The phone line infrastructure will operate such that there will be a total of 4 lines open at all times, meaning the VOR service can accommodate 4 calls simultaneously. Given the nature of the supervision, operators should be able to place participants on hold to either answer a new incoming call or to toggle between calls during the 30-minute supervision period. Each operator will be responsible for tracking by hand which participants are on which line so as to ensure they provide support to the right individuals. All papers or documents created for the purpose of tracking multiple calls will be destroyed immediately prior to the end of an operator’s shift; this does not include the call logs which will be entered into the database on a regular basis.

Because the investigators are building a phone line with the ability to accommodate 4 active lines simultaneously, they will not leave participants unanswered or on hold for any length of time. Instead, if they cannot immediately reach an operator, the call will go to voice mail. This protocol and subsequent software were co-designed with our PWLE advisory group, TELUS (providing in-kind technology support) and the research team.

### Recruitment

The site of the study and location of recruitment will be Calgary, Alberta. Study personnel will share details of the study with the following organizations and representatives who make up the advisory team: chapter of Alberta Addicts Who Educate and Advocate Responsibly (AAWEAR), Safeworks (who provide some of the harm reduction in Calgary), the SORCe (community resource center in Calgary), HIV Community Link (a local harm reduction nonprofit organization), and the Calgary Canadian Mental Health Association.

The organizations will be asked if they are willing to share information about the study with clients. Interested participants will be able to contact the study team directly or have the organization’s representative contact the study team on their behalf (to simplify the process for the potential participant).

Recruitment commenced in June 2020 and was paused at government request in June 2020. Recruitment is designed to be paused at 4 participants until there have been 15 calls to the phone line. This will allow for changes to the phone line if necessary (due to high volumes at certain times, etc).

The eligibility criteria for clients are the following: able to give informed consent (ie, able to speak and understand English and be over 18 years of age); admission of using opioids nonmedically, including using illicit opioids, using prescription opioids without a current prescription, using doses greater than those prescribed, or using opioids recreationally; access to a phone line in the location they primarily use opioids (this can be a landline or a cell phone); and a resident of Calgary. Meanwhile, the client exclusion criteria are as follows: unable to give informed consent (ie, unable to understand English, under 18 years of age, or otherwise legally unable to give consent), exclusive medical use of opioids, no access to a phone, or not a resident of Calgary.

For peer operators, the eligibility criteria are the following: able to give informed consent (ie, able to speak and understand English and be over 18 years of age) and currently employed as a peer operator for the VOR study. Meanwhile, the exclusion criteria for peer operators are the following: unable to give informed consent (ie, unable to understand English, under 18 years of age, or otherwise legally unable to give consent) or not employed as a peer operator for the VOR study.

Peer operators were hired with a job posting that prioritized individuals with lived experience of substance use and a working knowledge of harm reduction and peer support practice. In all, 10 interviews were conducted, and 7 individuals were hired in late March 2020 so as to ensure sufficient coverage of the phone lines.

Each peer operator participated in over 20 hours of training that included a combination of online (zoom) and in person sessions throughout April, May, and June of 2020. Two local Calgary, Alberta agencies partnered to provide virtual training that covered a range of topics necessary for the project’s success, including theory and practice of peer support, recovery-oriented treatment, harm reduction, and phone-based crisis intervention. The research coordinator (SB) and primary investigator (KR) led training on the clinical trial’s standard operating procedures and how to use the phone technology.

Further to the training detailed above that we believe helps mitigate harms to individuals working as peer operators, SB, KR, and physician coleads (MT and SG) were available to peer operators for additional support and counsel after difficult calls or shifts. In case a peer operator could not reach supervising staff immediately, they would fill in a post-shift survey that would notify the research team that follow-up with a peer operator was needed. Finally, as an added precaution, it was arranged that peer operators could reach out to Calgary’s Distress Centre supervisors for immediate support in case of serious emotional distress.

### Enrolment Visit

#### Client

Normally, the enrolment visit would take place where the potential participants are most comfortable, with preference to the organization that they normally access. However, due to COVID-19 safety precautions, study staff will engage with potential participants only once a screening has taken place and an agreed upon outdoor public space is chosen.

During the enrolment visit, the study personnel will review the information sheet and consent form with the potential participant and screen for eligibility. If the person is eligible and consents to participate, the study personnel will complete the baseline data collection survey.

After the survey is complete, the study personnel will review the information about the phone line and service with the participant and provide the information sheet with the number and reinforce that they should call the line if they are using opiates alone.

#### Peer Operator

Prior to the first shift on the phone lines, each peer operator will be contacted by KR to discuss participation in the research trial (registered with ClinicalTrials.gov; NCT04391192). The information sheet will be discussed, and consent offered. They will be told they can revoke consent at any time and that participation will not affect their employment.

### Service Usage

#### Client

Participants will dial in to the service number (toll free) when they are using opiates alone. The PWLE operator will follow the call flow (Multimedia Appendix 1). The PWLE operator will record caller information during the call (Multimedia Appendix 2). There is no limit to the number of times a participant can use the service during their enrolment, each call will be identified by date and time.

#### PWLE Operator

After each shift, there will be a short survey for each PWLE operator to complete regarding the operator’s experience during that shift. The survey will be part of the job to ensure operators are supported appropriately; however, those PWLE operators who have given consent will have these surveys included in the analysis of the study.

Although an overdose during a call is not considered an adverse event and is rather the purpose of the intervention, there will be ongoing monitoring of the resultant 911 dispatch and continual work to ensure appropriate follow-up. The weekly interviews of clients and monitoring of the administrative database to identify opioid overdose emergency visits that occur without use of the intervention may result in information that could be considered adverse events and will be investigated by the research team to understand the context and implications for the project.

### Interviews

#### Client

The participants will all be interviewed weekly by study personnel to gather information on the service (Multimedia Appendix 3).

If administrative data show that a participant attended an emergency department in Alberta or EMS was dispatched to them for an opioid poisoning, the participant will be contacted within a week and interviewed regarding why they did not call the service. The participants will explicitly consent to the study team monitoring administrative data for this purpose.

#### PWLE Operators

The PWLE operators will be interviewed weekly (Multimedia Appendix 4) during the study. These interviews will focus on the impact on the PWLE operator and process impact.

### Ethical Considerations

This study is to be conducted according to International standards of Good Clinical Practice (International Conference on Harmonization guidelines), the Declaration of Helsinki (2008 amendment, Seoul, Korea), applicable government regulations, and institutional research policies and procedures.

This protocol and any amendments have been submitted to a properly constituted independent ethics committees at the University of Alberta, known as the Health Research Ethics Board – Health Panel (HREB), and the University of Calgary, known as the Conjoint Health Research Ethics Board (CHREB), and are in agreement with local legal prescriptions for formal approval of the study conduct. The HREB has given approval under the study ID, Pro00088754, and the CHREB has given approval under the study ID, REB20-1043.

All participants for this study will be provided a consent form describing this study that contains sufficient information for participants to make an informed decision about their participation in this study. This consent form, along with all study materials, has been approved by the HREB. The formal consent of a participant, using the HREB-approved (Pro00088754) and CHREB-approved (REB20-1043) consent form, must be obtained before that participant undergoes any study procedure, and the consent form must be completed by the participant.

### Data Collection and Analysis Plan

Collection of all data will be compliant with the Health Information Act of Alberta [25]. Identifiers collected will include the Alberta Health Care number, birth year, and address for the location of each call to the service. All data will be collected originally on paper, transferred into electronic form by the study personnel, and then kept on an encrypted, password-protected laptop. The paper forms will be held in a locked filing cabinet in an office in a secured building.

### Analysis Plan

This is a phase 1 study designed to show the feasibility of a peer operator staffed virtual overdose response service, and thus there are no hypotheses being statistically tested.

Interview responses from both recruited participants and peer operators will be analyzed for common themes and any responses that demonstrate a safety concern. Safety concerns will be addressed as soon as they are identified. The interviews will be transcribed and entered into NVivo (QSR International) for coding and theme identification. Analysis will highlight barriers and facilitators to use of the intervention.

Process information will be collected and analyzed for trends in time of call, length of call, location of the participant (consistency), outcome of call EMS call outs, results of EMS call outs, and number of overdoses responded to.

### Study Withdrawal and Completion Rates

Any participant can withdraw at any time for any reason, and no further data will be gathered; however, data gathered to that point will not be removed from analysis. Peer operators can withdraw from the study without resigning from the position at any time; however it will be a qualified withdrawal where the previous data are included in the study. 

### Statistical Plan

All statistics will be descriptive. The investigators will report on how many times each participant calls the line, the time of the calls, and the outcome of the calls.

### Confidentiality

Information about study participants will be kept confidential and managed according to the requirements of the Health Information Act of Alberta. These regulations require a signed consent informing the participant of the following: What protected health information will be collected from participants in this study? Who will have access to that information and why? Who will use or disclose that information? What rights does the participant have to revoke authorization for use of protected health information?

In the event that a participant revokes authorization to collect or use protected health information, the investigator, KR, by regulation, will retain the ability to use all information collected prior to the revocation of participant authorization.

This study is investigating a novel intervention in a group that can be highly marginalized; therefore, we have formed an advisory council of people with lived experience to provide direction and interpretation of results.

## Results

The study was funded in 2019, enrolment opened in June of 2020 and paused in June of 2020. The intervention and study received significant community support, suggesting that it is an acceptable intervention; however, there are no study results available at this time.

## Discussion

Due to the physical distancing requirements currently in place due to COVID-19, many organizations are considering novel, virtual health care services. The timing of this study, which will provide vital information about providing overdose response to people who use substances alone, could not be better. This model of care does not replace the care and services provided in physical supervised consumption services, where clients can receive sterile supplies, overdose response kits, and some health care. However, this type of service is low barrier and innovative and may reach people who will not or cannot use physical supervised consumption services due to a myriad of reasons.

## Appendix

Multimedia Appendix 1 Call flow for VOR participant and nonparticipant calls.

Multimedia Appendix 2 Call logs for participant calls, completed by VOR operators.

Multimedia Appendix 3 Participant interview guide.

Multimedia Appendix 4 Operator interview guide.

## Abbreviations

AAWEAR: Alberta Addicts Who Educate and Advocate Responsibly
ACCH: Alberta Community Council on HIV
CHREB: Conjoint Health Ethics Research Board
CONSORT: Consolidated Standards of Reporting Trials
EMS: emergency medical services
HREB: Health Research Ethics Board
PWLE: people with lived experience
SCS: supervised consumption services
WHO: World Health Organization
VOR: virtual overdose response
