# Supplementary figures and images for: Virtual Overdose Response for People Who Use Opioids Alone: Protocol for a Feasibility and Clinical Trial Study
Source: JMIR Res Protoc. 2021 May 12;10(5):e20183. doi: 10.2196/20183 (PMC8156128; doi:10.2196/20183)

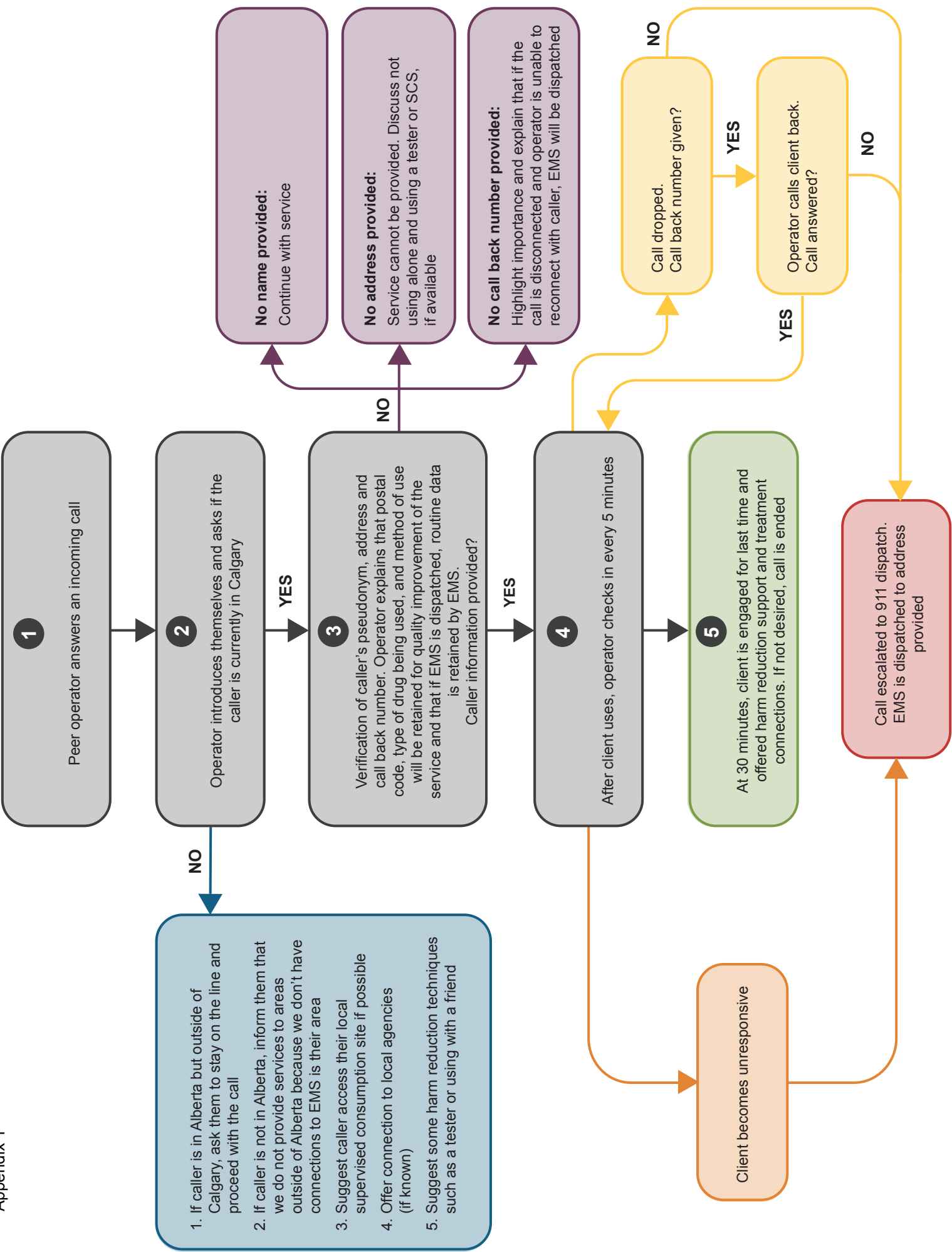

Supplement: Multimedia Appendix 1 [file resprot_v10i5e20183_app1.pdf]
