# Supplementary material for: Virtual Overdose Response for People Who Use Opioids Alone: Protocol for a Feasibility and Clinical Trial Study
Source: JMIR Res Protoc. 2021 May 12;10(5):e20183. doi: 10.2196/20183 (PMC8156128; doi:10.2196/20183)
Supplement: Multimedia Appendix 2 [file resprot_v10i5e20183_app2.pdf]

Virtual Overdose Response

Appendix 2

Virtual Overdose Response Call Log

Log number:

Date: **Time:**

Participant name: **Operator name:**

Address details provided:

Phone number provided:

Did you answer the call?

☐ Yes ☐ No (if no, complete details below)

Time of message listened to:

Time called back:

Call answered: ☐ Yes – Log number:

☐ No

Drug being used:

Method of use: **Time of use:**

Is client using new supplies?

☐ Yes ☐ No (if no, provide advice on where they can get supplies)

Does client want connection to services?

☐ Yes – Services suggested:

☐ No

Check in (total of 6 times):

1. Did participant respond? ☐ Yes ☐ No

Time:

2. Did participant respond? ☐ Yes ☐ No

Appendix 2

Time:

3. Did participant respond? ☐ Yes ☐ No

Time:

4. Did participant respond? ☐ Yes ☐ No

Time:

5. Did participant respond? ☐ Yes ☐ No

Time:

6. Did participant respond? ☐ Yes ☐ No

Time:

Was 911 dispatched?

☐ Yes – Time of dispatch:

☐ No

911 Event Number:

#__________

Notes from call:
