# Supplementary material for: Virtual Overdose Response for People Who Use Opioids Alone: Protocol for a Feasibility and Clinical Trial Study
Source: JMIR Res Protoc. 2021 May 12;10(5):e20183. doi: 10.2196/20183 (PMC8156128; doi:10.2196/20183)
Supplement: Multimedia Appendix 3 [file resprot_v10i5e20183_app3.pdf]

Appendix 3

Participant Interview Guide

The purpose of this interview is to get your opinion of the virtual supervised consumption service. This is the phone line that people can call if they are planning to use drugs alone. As a reminder, everything you say to me is confidential. Is it okay if I record the interview to ensure I record your answers right?

[TURN ON AUDIO RECORDER]

Topic Area I: Service usability
Question 1: Did you call the line this past week?
[IF YES – skip to topic area II]
[IF NO] – Question 2: did you use alone this past week?
[IF NO] – That's great. Can I help you with services or questions?
[IF NO] – end interview
[IF YES] – topic area I

Questions	Possible probes
Can you tell me about when you used alone this past week?	Where were you?<br>What time was it?<br>Why were you alone?
What happened that you didn't call the line?	Did you have your phone?<br>Did you have calling minutes?<br>Did you think it would take too long?<br>Were you worried the operator would judge you?<br>You weren't worried about anything bad happening, such as overdosing?
What could be done to make you more willing to call the line?	Specific examples<br>Probe for rationale behind suggestions
Did anything unusual happen?	Did you overdose?

[END OF INTERVIEW]

Topic Area II: Experiences using the line

Questions:	Possible probes:
IF patient answered YES: Can you tell me about the last time you called the service?	How did it go?<br>How did you feel?<br>Did you have any problems?
Has having the option to the phone line affected your health (if at all)?	Positives?<br>Negatives
Do you have any concerns or issues with the phone line (if any)?	Specific examples/incidents
What are some things you like about the phone line (if anything)?	Specific examples/incidents
What could be done better or what would you change about the phone line?	Specific examples<br>Why do you suggest that?
Question U: Did you use alone this past week and not call the line?	
[if yes to question U] can you tell me how many times you used alone without calling the line and how many times you did call?	
[if yes to question U – go to topic area I]	
Is there anything else you would like to say about the phone service?	

[END OF INTERVIEW]
