# Supplementary material for: Virtual Overdose Response for People Who Use Opioids Alone: Protocol for a Feasibility and Clinical Trial Study
Source: JMIR Res Protoc. 2021 May 12;10(5):e20183. doi: 10.2196/20183 (PMC8156128; doi:10.2196/20183)
Supplement: Multimedia Appendix 4 [file resprot_v10i5e20183_app4.pdf]

Appendix 4

Peer Operator Interview Guide

The purpose of this interview is to get your opinion of the virtual supervised consumption service. This is the phone line that people can call if they are planning to use drugs alone. As a reminder, everything you say to me is confidential. Is it okay if I record the interview to ensure I record your answers right?

[TURN ON AUDIO RECORDER]

**Questions:**	**Possible probes:**
Have you had any calls this week on your shift?<br>[IF YES go on with the rest of the questions]	Did you work this week
Can you describe your experience with the calls?	Any calls that went really well?<br>Any calls that didn't go well?
Did you have any problems with any calls?	Anyone not willing to give their address and phone number?<br>Any complaints from people on the line?
Did you respond to any non-responsive callers this week?<br>[IF YES go on – IF NO end interview]	Yes/No
Can you describe your experience with responding to the non-responsive caller?	How was interacting with 911 dispatch?<br>Did you hear any follow up afterwards?<br>Who did you hear from?
How might the service be improved?	Specific examples
Is there anything else you would like to tell me about your experience with the phone line?	

[END OF INTERVIEW]
